# Selective inhibitors of nuclear export: potential therapeutics for AR variant-expressing prostate cancer

**DOI:** 10.18632/oncotarget.26296

**Published:** 2018-11-09

**Authors:** Emmanuel S. Antonarakis

**Affiliations:** Department of Oncology, Sidney Kimmel Comprehensive Cancer Center at Johns Hopkins, Baltimore, MD, USA

**Keywords:** AR-V7, prostate cancer, selective inhibitors of nuclear export, selinexor, eltanexor

Designing therapies capable of targeting all forms of canonical and aberrant androgen receptor (AR) signaling (including AR overexpression/amplification, activating AR mutations, and constitutively-active AR splicing variants) remains one of the holy grails of advanced prostate cancer treatment [1]. In particular, treatment of castration resistant prostate cancer (CRPC) driven by the expression of AR splice variants such as AR-V7 has been a difficult challenge. With the exception of taxane chemotherapies [2] and perhaps immune checkpoint blockade strategies [3] which may be efficacious in a subset of patients with AR-V7–expressing prostate cancer, the majority of attempts to treat this lethal form of the disease have largely failed thus far [4]. Clearly, management of AR-V7–positive metastatic CRPC thus continues to represent an unmet medical need.

To try to address this unmet need, Aboukameel et al. [5] recently reported on a preclinical study employing selective inhibitors of nuclear export (SINE agents) as potential therapeutics for prostate cancers driven by aberrant AR signaling including AR splice variants. The authors focused their efforts on targeting exportin-1 (XPO1), a nuclear export protein involved in the transportation of macromolecules including tumor suppressor proteins (p53, Rb, p73, BRCA1, p21, p27, APC, SMAD4 and FOXO transcription factors) from the nucleus to the cytoplasm [6]. In previous studies, increased expression of XPO1 in prostate cancer was associated with high Gleason scores and an elevated risk of bone metastases [7]. In the current study, Aboukameel et al. demonstrated that increased expression of XPO1 was associated with higher AR-V7 levels, that disruption of XPO1 (through RNA interference or pharmacologic inhibition using SINE compounds: selinexor and eltanexor) inhibited expression of AR and AR-V7 together with abrogation of their transcriptional programs, and that inhibition of XPO1 *in vitro* and *in vivo* in AR-V7– expressing cell lines and xenograft models (either using SINE agents alone or in combination with abiraterone and enzalutamide) restricted tumor growth [5]. The authors concluded that XPO1 inhibition by selinexor or eltanexor might represent a promising treatment strategy for the management of human CRPC.

While this suggestion is theoretically attractive, the reality of XPO1 inhibition using clinical-grade SINE compounds in advanced prostate cancer patients has been somewhat challenging. To this end, a recent clinical trial testing selinexor in a population of metastatic CRPC patients who had failed at least one novel hormonal therapy (abiraterone or enzalutamide, or both) was terminated early after only 14 of 56 planned patients had enrolled, due to excess toxicity [8]. Interestingly, selinexor did show modest clinical activity in a subset of these patients: 2 of 14 men achieved a 50% PSA response (although neither PSA response was very durable), and 2 of 8 patients with measurable soft-tissue disease demonstrated an objective tumor response (although again these objective responses were short-lived). Interestingly, both of the patients who achieved PSA responses had previously received abiraterone as well as enzalutamide, suggesting that they had a high theoretical chance of expressing the AR-V7 splice variant, although this was not measured in the clinical trial. Ultimately, the trial was closed early because 5 of 14 patients (36%) experienced grade 3-4 treatment-related adverse events including anorexia, nausea, weight loss, fatigue and thrombocytopenia; three patients (21%) came off study due to unmanageable toxicity.

A second-generation SINE agent, eltanexor, is currently in clinical development and appears to have a more favorable toxicity profile than its predecessor. To this end, the ongoing phase 1/2 trial using eltanexor (NCT02649790) is targeting enrollment of patients with metastatic CRPC in addition to other cancer types (metastatic colorectal cancer, multiple myeloma and myelodysplastic syndrome). If acceptable safety and encouraging efficacy are observed in this trial, then future phase 2 studies should investigate not only the single-agent efficacy of eltanexor in metastatic CRPC but also its potential to induce synergy with enzalutamide and abiraterone, as suggested by the preclinical data. The results of these studies using eltanexor are eagerly awaited.
